# Ageing and agency: age-related changes in susceptibility to illusory experiences of control

**DOI:** 10.1098/rsos.161065

**Published:** 2017-05-24

**Authors:** Maria Cristina Cioffi, Gianna Cocchini, Michael J. Banissy, James W. Moore

**Affiliations:** Psychology Department, Goldsmiths University of London, Lewisham Way, London SE14 6NW, UK

**Keywords:** ageing, consciousness, volition, interoception, proprioception

## Abstract

Sense of agency (SoAg) is the feeling of control over one's actions and their effects. It can be augmented or attenuated by internal signals and by external cues. Research has shown a reduction in the SoAg in older adulthood, but the reasons behind this change remain unclear. We investigated agency processing differences that may underpin age-related changes in SoAg. Using a modified version of a vicarious agency paradigm, we tested the modulation of SoAg by manipulating external situational agency cues in younger and older adults. Our results show that the illusion of vicarious agency was less pronounced in older adults. These results were replicated in a second experiment which also showed that older adults performed significantly better in interoception and proprioception tasks. We suggest that increased reliance on internal cues may explain differences in agency processing in older adulthood.

## 1. Introduction

Sense of agency (SoAg) refers to the experience of initiating and controlling action in order to influence events in one's environment. It is inextricably linked to everyday notions of freedom and responsibility [1] and therefore forms an integral part of our cognitive and social lives [2].

Research suggests a general reduction in feeling of control over life events in older adulthood (e.g. [3–5]). Recent research also shows more specific age-related changes in SoAg, with older adults shown to be less sensitive to external manipulations of their sense of control [6]. Given the psychological and social significance of SoAg [7], these age-related changes warrant further scrutiny. This is something we aim to address in this study by examining the *neurocognitive* factors underpinning age-related changes in the SoAg.

In terms of the neurocognitive origins of SoAg, there have, traditionally, been two competing views. Some have suggested that SoAg arises principally from *internal* processes serving motor control [8,9]. On the other hand, *external* situational cues have been emphasized [10,11]. However, it is now becoming increasingly clear that this is a false dichotomy and that various cues contribute to SoAg [2,12–15]. These cues include internal sensorimotor signals and external situational information [2].

Moreover, Metcalfe *et al.* [6] showed that people's metacognition of agency changes across the lifespan. In particular, they showed that older adults were less sensitive to external performance manipulations, such as the insertion of temporal or spatial distortion between their movement and a cursor moving on the screen. Although not a direct test of this, Metcalfe *et al*.'s study suggests that the influence of external agency cues may be reduced in older adults. In the present experiments, we directly investigate the age-related changes in the influence of internal and external agency cues. We do so by testing the susceptibility to illusory experience of control induced by the manipulation of external agency cues.

## 2. Experiment 1

**Figure 1.**
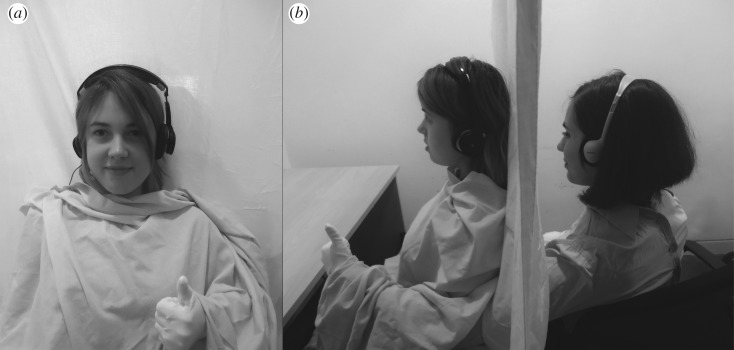
Experimental set-up (based on Wegner *et al*. [14]). Panel (*a*) shows what the participant sees in the mirror placed in front of her. (*b*) The set-up from the side, with the experimenter sitting behind the participant and putting her hand forward so that it appears where the participant's hand would normally be.

To investigate the influence of external agency cues in older adults we used the so-called ‘vicarious agency' paradigm developed by Wegner *et al.* [14] in a modified version we have previously used [16]. In this paradigm participants view themselves in a mirror while an experimenter stands behind them, hidden from view. The experimenter's hands are extended on each side of the participant in a body-congruent posture (see figure 1 for experimental set-up). Participants also wear headphones on which are played action previews. These previews are either congruent or incongruent with actions subsequently made by the experimenter. Wegner *et al*. found that the participants' experience of controlling these movements was increased when the previews were congruent with the action the experimenter made.

In this paradigm internal sensorimotor signals are absent as the participant remains stationary throughout the procedure. As such, this paradigm neatly demonstrates the influence of external situational cues (action previews and experimenter-made movements) on the SoAg. These cues can lead people to experience an SoAg over movements that are not their own. Given the indication that older adults seem less sensitive to external agency cues [6], we predicted that the magnitude of the illusory experience of (vicarious) agency in older adults would be reduced.

Sense of ownership (SoO) refers to the feeling that a body part is one's own and it is another core feature of self-awareness closely related to the SoAg [17]. In the light of this, we also predicted that older adults would experience less ownership on this task.

### 2.1. Method

#### 2.1.1. Participants

Two groups of participants were recruited. The ‘younger adult' group consisted of 14 participants (six females; average age = 23.0 years, s.d. = 4.7, range = 17–34). The ‘older adult' group consisted of 14 participants (six females; average age = 64.2 years, s.d. = 6.2, range = 54–72).

The sample size for our experiments were guided by previous studies using the vicarious agency paradigm (e.g. *n* = 16 in Wegner *et al*. [14]; *n* = 8 in Cioffi *et al*. [16]). Our sample size exceeded these previous studies but was constrained by the availability of older adult participants within the time frame of this project.

#### 2.1.2. Procedure

Participants sat on a chair facing a full-length mirror at a distance of 1 m. Participants wore over-ear headphones on which were played the action previews. A blue sheet covered the participants' body from the shoulders downwards and a blue curtain was placed behind their back to block their view of the experimenter (figure 1).

Participants' arms were placed out of view under the sheet. The experimenter put on another set of headphones to hear the instructions, a blouse that was the same colour as the sheet covering the participant and a pair of white gloves. The experimenter was positioned behind the curtain. The experimenter placed their arm (either left or right) forward through two specific holes in the curtain, so that it appeared where the participant's own arm would have been. Participants were asked to look at the mirror in front of them while the experimenter performed the gestures with either the left or the right hand and to remain still during the experiment.

A tape with a list of 16 unimanual action instructions was played (e.g. ‘make a waving gesture', ‘snap the fingers twice', ‘point to the mirror’). The examiner performed an action immediately after each instruction. Each instruction–action stimulus, consisting of one instruction plus action performed by the experimenter, lasted between 8 and 10 s with a 3 s break between stimuli.

There were two within-subject conditions. In the *match condition*, the action corresponded to the instruction, whereas in the *mismatch condition* each instruction was randomly matched with a different action (e.g. after the instruction ‘make a waving gesture' the examiner snapped their fingers). In this mismatch condition, the gesture was different for every repetition of the same instruction (e.g. on the second repetition, after the instruction ‘make a waving gesture’ the examiner pointed to the mirror). These conditions were completed for both the right and left hand separately. The order of match‐mismatch conditions and the order of hand tested were counterbalanced across participants. The list of 16 instruction–action stimuli was repeated from the beginning to the end without interruption three times for each of the four conditions (match/mismatch and left/right hand), so as to augment the effects of this manipulation. The list of 16 action instructions was kept identical for the entire experiment, only the actions performed by the experimenter changed accordingly to the condition.

At the end of each condition participants were asked to report their experiences by answering three questions on a 7-point scale from 1—not at all to 7—very much (this was done for each hand and the judgements were averaged across hands). In total, each participant was given 12 instruction–action list repetitions (i.e. three repetitions for each of the four conditions) and provided four ratings for each of the questions reported below.

The questions were similar as those included in Wegner *et al*.'s [14] study.

Anticipation: *To what degree did you feel you could anticipate the movements of the arm?*
This control question assesses the success of the manipulation and whether the primes were attended to. This was included because a failure to attend to the primes may explain any putative performance differences in the two groups. If primes are attended to then anticipation judgements should be higher in the match than in the mismatch conditions. This question also serves as a control for any response bias (e.g. general tendency of one group to report higher or lower ratings).
SoAg: *How much control did you feel you had over the arm's movements?*
This target question directly assesses the experience of agency.
SoO: *To what degree did the arm feel like it belonged to you?*
This question provides an additional measure of the effect of the manipulation, examining the impact on SoO over the body part.

A practice session consisting of three match and three mismatch trials was performed at the beginning of the experiment.

### 2.2. Results

#### 2.2.1. Anticipation

Anticipation was measured by asking participants ‘To what degree did you feel you could anticipate the movements of the arm?’

**Figure 2.**
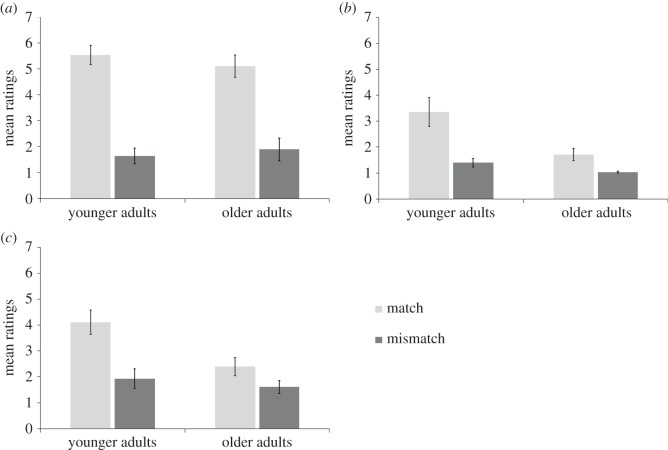
Mean ratings for anticipation (*a*), agency (*b*) and ownership (*c*) in match and mismatch conditions for experiment 1. The error bars show standard deviation across participants. Older adults show a weaker effect of agency and owenership illusions.

Figure 2*a* shows mean anticipation judgements in the ‘younger adult' and ‘older adult' groups plotted as a function of instruction–action congruence. A 2 × 2 mixed-design ANOVA was performed on the mean anticipation judgements (between-subjects factor: ‘age' young/old, within-subjects factor: ‘congruence' match/mismatch). We found a significant main effect of congruence (*F*_1,26_ = 85.56, *p* < 0.001, $\eta_{\mathrm{partial}}^{2}=0.77$). Participants reported greater anticipation in the match condition, where the gesture corresponded to the listened instruction, than in the mismatch condition. Crucially, there was no main effect of ‘age’ (*F*_1,26_ = 0.05, *p* > 0.250, $\eta_{\mathrm{partial}}^{2}=0.01$) and no significant interaction between age and congruence (*F*_1,26_ = 0.78, *p* > 0.250, $\eta_{\mathrm{partial}}^{2}=0.03$). Overall, these results show that the primes and actions were equally well attended to in each group.

#### 2.2.2. Sense of agency

The SoAg was measured by asking participants ‘How much control did you feel you had over the arm's movements?'

Figure 2*b* shows mean agency judgements in the ‘younger adult' and ‘older adult' groups plotted as a function of prime-action congruence. A 2 × 2 mixed-design ANOVA was performed on the mean agency judgements (between-subjects factor: ‘age’ young/old, within-subjects factor: ‘congruence' match/mismatch). We found a significant main effect of congruence (*F*_1,26_ = 18.02, *p* < 0.001, $\eta_{\mathrm{partial}}^{2}=0.41$). Participants reported having a stronger experience of control over the experimenter's arm movements in the match condition than in the mismatch condition. There was also a significant main effect of ‘age' (*F*_1,26_ = 9.81, *p* = 0.004, $\eta_{\mathrm{partial}}^{2}=0.27$), showing that the overall SoAg was significantly weaker in the older adults. Crucially, there was a significant interaction between ‘age' and ‘condition', (*F*_1,26_ = 4.26, *p* = 0.049, $\eta_{\mathrm{partial}}^{2}=0.14$). Inspection of figure 2*b* suggests that the effect of the experimental manipulation was weaker in older adults. Two *post hoc t*-tests were carried out to examine this interaction (Bonferroni's correction: *p* < 0.025). Although there was a significant effect of congruence in both age groups, this effect was weaker in older adults (‘younger adults', match versus mismatch, match: *M* = 3.36, mismatch: *M* = 1.39, mean difference = 1.97, 95% CI = [0.72, 3.21], *t*_13_ = 3.41, *p* = 0.005, *d* = 1.28; ‘older adults’, match versus mismatch, match: *M* = 1.71, mismatch: *M* = 1.04, mean difference = 0.67, 95% CI = [0.16, 1.19], *t*_13_ = 2.85, *p* = 0.014, *d* = 1.03). These findings suggest that the vicarious agency illusion was attenuated in older adults.

#### 2.2.3. Sense of ownership

The SoO was measured by asking participants ‘To what degree did the arm feel like it belonged to you?'

Figure 2*c* shows mean ownership judgements in the ‘younger adult' and ‘older adult' groups plotted as a function of prime-action congruence. A 2 × 2 mixed-design ANOVA was performed on the mean ownership judgements (between-subjects factor: ‘age’ young/old, within-subjects factor: ‘congruence' match/mismatch). We found a significant main effect of congruence (*F*_1,26_ = 22.16, *p* < 0.001, $\eta_{\mathrm{partial}}^{2}=0.46$). Participants reported a stronger experience of ownership in the match condition than in the mismatch condition. There was also a significant main effect of ‘age' (*F*_1,26_ = 5.88, *p* = 0.023, $\eta_{\mathrm{partial}}^{2}=0.18$) suggesting that the overall experience of ownership was weaker in the older adults. Moreover, there was a significant interaction between ‘age' and ‘condition' (*F*_1,26_ = 4.89, *p* = 0.036, $\eta_{\mathrm{partial}}^{2}=0.16$). Inspection of figure 2*c* suggests that the effect of the experimental manipulation was weaker in older adults. Two *post hoc t*-tests were carried out to examine this interaction (Bonferroni's correction: *p* < 0.025). These tests show that there was only a significant effect of congruence on ownership in the younger adults (‘younger adults', match versus mismatch, match: *M* = 4.11, mismatch: *M* = 1.93, mean difference = 2.18, 95% CI = [1.01, 3.35], *t*_13_ = 4.03, *p* = 0.001, *d* = 1.36; ‘older adults’, match versus mismatch, match: *M* = 2.39, mismatch: *M* = 1.61, mean difference = 0.78, 95% CI = [0.09, 1.48], *t*_13_ = 2.44; *p* = 0.030, *d* = 0.78). These findings mirror the agency effects reported above and confirm that the older adults were less sensitive to the vicarious agency illusion.

### 2.3. Discussion

The vicarious agency paradigm developed by Wegner *et al.* [14] demonstrated the influence of external situational cues on SoAg. Using this paradigm we were able to directly investigate the modulation of SoAg by external agency cues in younger and older adults. We replicated the vicarious agency effect in a group of younger and older adults, with both groups presenting higher ratings in the match conditions compared to mismatch conditions in the SoAg and the SoO. Moreover, and in line with our initial predictions, it was found that this illusion of vicarious agency was less pronounced in older adults, suggesting a weaker influence of external agency cues. Similar findings were observed when we measured SoO, which is an aspect of self-awareness closely related to SoAg. As both groups did not show any difference in the check question, i.e. the anticipation question, we can conclude that the group differences in agency and ownership on this task are unlikely to be explained by differences in attention, or any response bias.

According to the cue integration framework (e.g. [2]), the reduction in the influence of external cues in older adults could be owing to a relative increase in the reliability of internal cues. This research question is addressed in a second experiment where additional measures of internal processes are considered.

## 3. Experiment 2

Given the relatively small sample size of experiment 1, one aim of this experiment was to replicate those results with a new sample. Another aim was to further investigate the possible mechanisms responsible for the changes in SoAg and SoO. The results of experiment 1 suggest that the influence of external cues on agency and ownership processing is attenuated in older adults. In this experiment, we examine the possibility that older adults tend to rely more than younger adults on internal cues, by investigating additional measures of interoceptive and proprioceptive accuracy. If our findings in older adults are owing to increased relative reliability of internal cues, older adults should show improvements in interoceptive and proprioceptive accuracy relative to younger adults.

### 3.1. Method

#### 3.1.1. Participants

The ‘younger adult' group consisted of 18 participants (six females; average age = 20.27 years, s.d. = 3.9, range = 18–35). The ‘older adult' group consisted of 17 participants (nine females; average age = 71 years, s.d. = 7.6, range = 62–92). All were naive to the tasks and did not take part in experiment 1.

#### 3.1.2. Procedure

The experiment included the vicarious agency illusion paradigm, and measures of proprioceptive and interoceptive accuracy. As with the first experiment, all measures assessed are reported in the paper and no interim statistical analysis was performed.

The procedure for the illusion of vicarious agency was identical to experiment 1.

As a measure of interoceptive accuracy, a heartbeat monitoring task was used [18]. Participants were asked to sit comfortably, keep their legs uncrossed and close their eyes. Their heartbeat was monitored by a pulse transducer attached to the participant's non-dominant index finger. They were instructed to silently count their own heartbeats during an interval of time that started and finished with an audio cue. At the end of each interval, each participant was asked to report the number of heartbeats counted. The six intervals were 20, 20, 30, 30, 40 and 40 s each, presented in a randomized order. The interoceptive accuracy was measured by calculating the proportional discrepancy between the perceived and the actual number of heartbeats: |counted heartbeats − recorded heartbeats|/recorded heartbeats. This was averaged across intervals to provide an accuracy error index, with 0 indicating no discrepancy between perceived and actual numbers of heartbeats, and larger discrepancies indicated by larger values.

Proprioceptive accuracy was measured using a task inspired by well-established contralateral concurrent matching tasks [19]. Participants were asked to sit in front of a desk and to familiarize themselves with a plain sheet of A3 paper. They were then asked to close their eyes and keep them closed until they were explicitly required to open them. An A3 sheet was then placed in front of them, centred on the participant's body, while the experimenter verbally explained this. The experimenter took the participant's right index finger and placed it on a preprinted dot. The participant was asked to place their left index finger such that it was mirroring their right index finger across the centre of the A3 sheet. The procedure was repeated for a total of six dots, three for each hand. The participant was asked to open their eyes only at the end of the six trials. The proprioceptive accuracy measure was calculated as the average of the discrepancy (in centimetres) between each actual mirrored position and the one indicated by the participant (0 = no discrepancy).

The three tasks were presented in counterbalanced order.

### 3.2. Results

#### 3.2.1. Vicarious agency paradigm

As in experiment 1, the vicarious agency paradigm was analysed with separate 2 × 2 mixed-design ANOVAs on the mean judgements for each question, with ‘congruence' the within-subjects factor and ‘age' (young/old) the between-subjects factor.

##### 3.2.1.1. Anticipation

**Figure 3.**
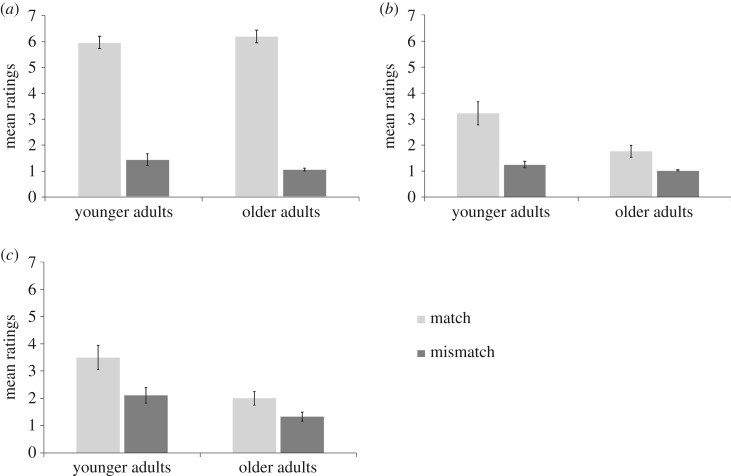
Mean ratings for anticipation (*a*), agency (*b*) and ownership (*c*) in match and mismatch conditions for experiment 2. The error bars show standard deviation across participants. Similarly to experiment 1, older adults show a weaker effect of agency and ownership illusions.

The results directly replicate those from experiment 1 (figure 3*a*). A main effect of congruence was found (*F*_1,33_ = 489.96, *p* < 0.001, $\eta_{\mathrm{partial}}^{2}=0.94$), showing that participants reported a greater feeling of anticipation in the match conditions compared with the mismatch conditions. Importantly, there was no main effect of age (*F*_1,33_ = 0.15, *p* > 0.250., $\eta_{\mathrm{partial}}^{2}<0.01$) and no interaction between congruence and agency (*F*_1,33_ = 2.00, *p* = 0.166, $\eta_{\mathrm{partial}}^{2}=0.06$). These results showed that the prime-action relationship was equally well attended to by both groups. Therefore, differences in attention are unlikely to explain differences in agency and ownership on this task.

##### 3.2.1.2. Sense of agency

The results directly replicate those from experiment 1. A main effect of congruence was found (*F*_1,33_ = 37.57, *p* < 0.001, $\eta_{\mathrm{partial}}^{2}=0.53$), showing that participants felt a stronger SoAg over the experimenter's arm in the match compared to the mismatch conditions. A main effect of age was also found (*F*_1,33_ = 7.91, *p* = 0.008, $\eta_{\mathrm{partial}}^{2}=0.19$), showing that the overall SoAg was lower in older adults. Crucially, a significant interaction between age and congruence was also found, (*F*_1,33_ = 7.84, *p* = 0.008, $\eta_{\mathrm{partial}}^{2}=0.19$). Inspection of figure 3*b* suggests that the effect of the experimental manipulation was weaker in older adults. Two *post hoc t*-tests were carried out to examine this interaction (Bonferroni's correction: *p* < 0.025). Although there was a significant effect of congruence in both age groups, this effect was weaker in older adults (‘younger adults', match versus mismatch, match: *M* = 3.22, mismatch: *M* = 1.25, mean difference = 1.97, 95% CI = [1.19, 2.75], *t*_17_ = 5.34, *p* < 0.001, *d* = 1.43; ‘older adults’, match versus mismatch, match: *M* = 1.76, mismatch: *M* = 1.03, mean difference = 0.73, 95% CI = [0.24, 1.23], *t*_16_ = 3.18, *p* = 0.006, *d* = 1.08). These results directly replicate those of experiment 1, showing that the experience of agency in older adults was not as strongly modulated by the experimental manipulation.

##### 3.2.1.3. Sense of ownership

The results directly replicate those from experiment 1. A main effect of congruence was found (*F*_1,33_) = 38.16, *p* < 0.001, $\eta_{\mathrm{partial}}^{2}=0.54$), showing that participants felt a stronger SoO over the experimenter's arm in the match compared to mismatch conditions. A main effect of age was also found (*F*_1,33_ = 8.15, *p* = 0.007, $\eta_{\mathrm{partial}}^{2}=0.20$), showing that the overall SoO was lower in older adults. Crucially, a significant interaction between age and congruence was also found, (*F*_1,33_ = 4.54, *p* = 0.041, $\eta_{\mathrm{partial}}^{2}=0.121$). Inspection of figure 3*c* suggests that the effect of the experimental manipulation was weaker in older adults. Two *post hoc t*-tests were carried out to examine this interaction (Bonferroni's correction: *p* < 0.025). Although there was a significant effect of congruence in both age groups, this effect was weaker in older adults (‘younger adults', match versus mismatch, match: *M* = 3.50, mismatch: *M* = 2.11, mean difference = 1.39, 95% CI = [0.79, 1.98], *t*_17_ = 4.93, *p* < 0.001, *d* = 0.88; ‘older adults’, match versus mismatch, match: *M* = 2, mismatch: *M* = 1.32, mean difference = 0.68, 95% CI = [0.31, 1.04], *t*_16_ = 3.95, *p* = 0.001, *d* = 0.79). These results directly replicate those of experiment 1, showing that the experience of ownership in older adults was not as strongly modulated by the experimental manipulation.

#### 3.2.2. Interoceptive and proprioceptive accuracy

**Figure 4.**
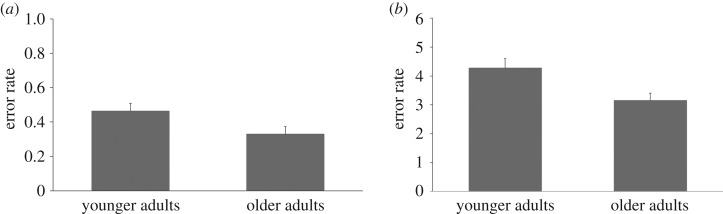
(*a*) Error rate of interoceptive accuracy, with 0 indicating no discrepancy between actual and estimated number of heartbeats. (*b*) Error rate of proprioceptive accuracy with 0 indicating no discrepancy between actual and estimated position. The error bars show standard deviation across participants. Older adults perform better in both tasks.

We compared the performance of the two age groups on the proprioceptive and interoceptive tasks (figure 4). We found that older adults performed significantly better than younger adults on both tasks (‘interoceptive error', younger adults versus older adults, younger adults: *M* = 0.47, older adults: *M* = 0.33, mean difference = 0.14, 95% CI = [0.02, 0.26], *t*_33_ = 2.21, *p* = 0.034, *d* = 0.80; ‘proprioceptive error', younger adults versus older adults, younger adults: *M* = 4.28, older adults: *M* = 3.16, mean difference = 1.12, 95% CI = [0.31, 1.94], *t*_33_ = 2.80, *p* = 0.009, *d* = 0.95). This result suggests that older adults monitor internal bodily signals more successfully than younger adults.

### 3.3. Discussion

The results of experiment 2 on the vicarious agency task replicated those of experiment 1, showing that the influence of external cues on agency (and ownership) is attenuated in older adults. Looking at proprioceptive and interoceptive accuracy, we found that older adults performed better on the proprioceptive and interoceptive tasks compared to younger adults. In line with the cue integration framework, this may help explain why the older adults are less sensitive to the illusion. Older adults would discount external information in favour of more reliable internal cues.

## 4. General discussion

The aim of this study was to investigate agency processing changes in older adulthood. Older adulthood is associated with marked changes in both the general feeling of control over life events (e.g. [3,20]) and also the more specific SoAg [6]. Moreover, these changes can have a strong effect on quality of life (e.g. [4]). So far few studies have directly considered the underlying neurocognitive mechanisms of these age-related changes. The two experiments presented here aim to address this oversight. Our findings suggest that agency processing in older adults is less sensitive to external cuing and that this may be linked to relative increase in the reliability of internal cues in older adults.

Although more work is needed to fully understand the mechanisms underpinning age-related changes in SoAg, our results offer some preliminary insights. Both of our experiments show that older adults were less sensitive to illusory agency experiences (both in terms of agency and ownership). At first glance, this may seem advantageous as it suggests a more robust and less malleable experience of agency in older adults. However, in the context of a more recent optimal cue integration model of SoAg [2,13], this may in fact point towards an information processing *deficit*, which in turn may be responsible for age-related changes in SoAg. More specifically, this cue integration approach emphasizes the contribution of various sources of information to SoAg. Moreover, it is thought that these different sources of information are optimally integrated; the dominance of internal or external cues to agency will depend on their relative reliability [2,13]. In this way, if there are compelling external cues to agency these will dominate (and similarly for internal cues). The fact that the experience of agency in older adults is less sensitive to compelling external cues suggests that they may be discounting potentially useful sources of information. As such, agency processing in older adults may not be optimal. Although speculative, this discounting might be linked to the overall reduction in SoAg associated with older adulthood—by not optimally integrating *different* sources of information older adults may be less likely to recognize their own causal efficacy.

Experiment 2 aimed to investigate possible mechanisms underpinning this effect. We measured the performance on two internal monitoring tasks: interoceptive and proprioceptive accuracy. We found that older adults performed significantly better on measures of proprioceptive and interoceptive accuracy. This may explain why the older adults were less susceptible to the illusion: an increase in the relative weighting of internal cues diminished the effect of external cues on agency processing in older adults.

In summary, our study aimed to investigate agency processing differences underpinning age-related changes in SoAg. Using Wegner *et al*.'s [14] vicarious agency paradigm our findings suggest that the SoAg in older adults is less influenced by external cuing. Furthermore, our findings also suggest that this may be linked to an increase in the relative reliability of internal cues in older adults. Although more work needs to be done, we suggest that this difference in agency processing may contribute to the overall reduction in SoAg in older adulthood.

## Data accessibility

All datasets supporting this article are available from the Dryad Digital Repository: http://dx.doi.org/10.5061/dryad.td8d3 [21].

## References

[1] Haggard P, Tsakiris M 2009 The experience of agency. Curr. Dir. Psychol. Sci. 18, 242–246. (doi:10.1111/j.1467-8721.2009.01644.x)

[2] Moore JW, Fletcher PC 2012 Sense of agency in health and disease: a review of cue integration approaches. Conscious Cogn. 21, 59–68. (doi:10.1016/j.concog.2011.08.010) 21920777 10.1016/j.concog.2011.08.010 PMC3315009

[3] Heckhausen J, Schulz R 1995 A life-span theory of control. Psychol. Rev. 102, 284–304. (doi:10.1037/0033-295X.102.2.284) 7740091 10.1037/0033-295x.102.2.284

[4] Langer EJ, Rodin J 1976 The effects of choice and enhanced personal responsibility for the aged: a field experiment in an institutional setting. J. Pers. Soc. Psychol. 34, 191–198. (doi:10.1037/0022-3514.34.2.191) 1011073 10.1037//0022-3514.34.2.191

[5] Rodin J, Langer EJ 1977 Long-term effects of a control-relevant intervention with the institutionalized aged. J. Pers. Soc. Psychol. 35, 897–902. (doi:10.1037/0022-3514.35.12.897) 592095 10.1037//0022-3514.35.12.897

[6] Metcalfe J, Eich TS, Castel AD 2010 Metacognition of agency across the lifespan. Cognition 116, 267–282. (doi:10.1016/j.cognition.2010.05.009) 20570251 10.1016/j.cognition.2010.05.009

[7] Moore JW. 2016 What is the sense of agency and why does it matter? Front. Psychol. 7, 1272 (doi:10.3389/fpsyg.2016.01272) 27621713 10.3389/fpsyg.2016.01272 PMC5002400

[8] Blakemore S-J, Wolpert DM, Frith CD 2002 Abnormalities in the awareness of action. Trends Cogn. Sci. 6, 237–242. (doi:10.1016/S1364-6613(02)01907-1) 12039604 10.1016/s1364-6613(02)01907-1

[9] Haggard P 2005 Conscious intention and motor cognition. Trends Cogn. Sci. 9, 290–295. (doi:10.1016/j.tics.2005.04.012) 15925808 10.1016/j.tics.2005.04.012

[10] Wegner DM 2002 The illusion of conscious will. Cambridge, MA: MIT Press.

[11] Wegner DM 2003 The mind's best trick: how we experience conscious will. Trends Cogn. Sci. 7, 65–69. (doi:10.1016/S1364-6613(03)00002-0) 12584024 10.1016/s1364-6613(03)00002-0

[12] Kranick SM, Hallett M 2013 Neurology of volition. Exp. Brain Res. 229, 313–327. (doi:10.1007/s00221-013-3399-2) 23329204 10.1007/s00221-013-3399-2 PMC4744643

[13] Moore JW, Wegner DM, Haggard P 2009 Modulating the sense of agency with external cues. Conscious. Cogn. 18, 1056–1064. (doi:10.1016/j.concog.2009.05.004) 19515577 10.1016/j.concog.2009.05.004

[14] Wegner DM, Sparrow B, Winerman L 2004 Vicarious agency: experiencing control over the movements of others. J. Pers. Soc. Psychol. 86, 838–848. (doi:10.1037/0022-3514.86.6.838) 15149258 10.1037/0022-3514.86.6.838

[15] Wegner DM, Sparrow B 2004 Authorship processing. In The cognitive neurosciences, (ed. M Gazzaniga), pp. 1201–1209, 3rd edn. Cambridge, MA: MIT Press.

[16] Cioffi MC, Banissy MJ, Moore JW 2016 ‘Am I moving?’ An illusion of agency and ownership in mirror-touch synaesthesia. Cognition 146, 426–430. (doi:10.1016/j.cognition.2015.10.020) 26550800 10.1016/j.cognition.2015.10.020

[17] Gallagher S 2000 Philosophical conceptions of the self: implications for cognitive science. Trends Cogn. Sci. 4, 14–21. (doi:10.1016/S1364-6613(99)01417-5) 10637618 10.1016/s1364-6613(99)01417-5

[18] Garfinkel SN, Seth AK, Barrett AB, Suzuki K, Critchley HD 2015 Knowing your own heart: distinguishing interoceptive accuracy from interoceptive awareness. Biol. Psychol. 104, 65–74. (doi:10.1016/j.biopsycho.2014.11.004) 25451381 10.1016/j.biopsycho.2014.11.004

[19] Goble DJ. 2010 Proprioceptive acuity assessment via joint position matching: from basic science to general practice. Phys. Ther. 90, 1176–1184. (doi:10.2522/ptj.20090399) 20522675 10.2522/ptj.20090399

[20] Lachman ME, Firth KM 2004 The adaptive value of feeling in control during midlife. In How healthy are we?: A national study of well-being at midlife (eds Brim OG, Ryff CD, Kessler R), pp. 320–349. Chicago, IL: University of Chicago Press.

[21] Cioffi MC, Cocchini G, Banissy MJ, Moore JW 2017 Data from: Ageing and agency: age-related changes in susceptibility to illusory experiences of control. Dryad Digital Repository. (doi:10.5061/dryad.td8d3) 10.1098/rsos.161065 PMC5451800 28572999

